# Ten-year data analysis of digestive system malignancies in Babol, North of Iran: 2008-2017

**DOI:** 10.22088/cjim.13.1.76

**Published:** 2022

**Authors:** Leili Sadeghi Amiri, Novin Nikbakhsh, Mostafa Javanian, Simin Mouodi, Tahere Mousavi, Sedigheh Alijanpour, Fattaneh Vala, Mostafa Mirzad, Javad Shokri Shirvani, Hoda Shirafkan

**Affiliations:** 1 Basic Science Department, Agricultural Science and Natural Resources University, Sari, Iran; 2 Cancer Research Center, Health Research Institute, Babol University of Medical Sciences, Babol, Iran; 3 Infectious Diseases and Tropical Medicine Research Center, Health Research Institute, Babol University of Medical Sciences, Babol, Iran; 4 Social Determinants of Health Research Center, Health Research Institute, Babol University of Medical Sciences, Babol, Iran; 5 Cancer Registry Department, Babol University of Medical Sciences, Babol, Iran; 6 Health Research Institute, Babol University of Medical Sciences, Babol, Iran

**Keywords:** Digestive System, Gastrointestinal neoplasms, Incidence, Registries

## Abstract

**Background:**

Unlike some regions of the world where digestive system cancers are not considered as important health problems, these neoplasms are among the most common malignancies in the northern region of Iran.

**Methods:**

This observational analytical study was carried out based on data collected by the Cancer Registration Center affiliated to the Vice Chancellery for Health of Babol University of Medical Sciences, North of Iran, during 2008-2017. Crude incidence rate (CR), and age-standardized incidence rate (ASR) have been calculated for different GI cancers, based on the primary involved site; and have been compared in different years, patients' age, gender and place of residence.

**Results:**

Totally, 4332 records were related to digestive system cancers. Mean age of patients was 63.48±14.73 years; men (2743; 63.3%) were more affected than women (1589; 36.7%) (p<0.001). The most incident malignancies of digestive system were from stomach, colorectal and esophagus in men; and colorectal, stomach and esophagus in women, respectively. These three cancers accounted for 3725 (85.98%) of total GI malignancies. The mean age of patients in various types of GI cancers was statistically different (p<0.001). Age- standardized incidence rate showed different values in different years; from 521.40 (95% CI: 462.79-580.00) in year 2016 to 1834.33 (95% CI: 1637.36-2031.29) in year 2008.

**Conclusion:**

Gastric, esophageal and colorectal cancers were the most prevalent digestive system malignancies in Babol, North of Iran, and accounted for about 86% of all GI tract cancers. A considerable variation has been found in incident gastrointestinal cancers in different years.

Cancer has been represented as the second leading cause of death in the world (1) and is estimated to have caused 9.6 million deaths in 2018 (2). According to the World Health Organization, the most common types of cancer are lung, prostate, colorectal, stomach, and liver in males; and breast, colorectal, cervix and thyroid in females (2). In Iran, cancer has been reported as the third most common cause of death after cardiovascular disease and car accident (3). The pattern of cancer in Iran is different from the developed countries; for example, gastric and esophageal cancers which are less common in the developed countries, are among the most common types of cancer in Iran (4). A systematic analysis for the global burden of 29 cancer groups in different countries revealed that the incidence rate of cancer in Iran is considerable (5). Digestive system cancers are a group of malignancies which involve the organs that food and liquids pass through when they are swallowed, digested, absorbed, and leave the body. 

These organs are included the mouth, pharynx, esophagus, stomach, small intestine, colon, rectum, anus, liver, biliary system, and the pancreas (6). It has been reported that the incidence and mortality rate of these cancers are increasing in the world, and in Asia (7). Among them, gastric, esophageal and colorectal malignancies are known as the three most common types of GI cancers (8). Cancer registry, as a systematic process to collect, store, analyze, and report the relevant data to cancer, is an important approach to identify the current situation of various malignancies in each region to implement proper interventional programs for prevention and control of different neoplasms, especially cancers that are more common or have an increasing trend over time (9). This approach has been established in the health care system of Iran for more than 20 years to distinguish the current situation of various cancers (10). 

Two strategies have been implemented to collect data related to the patients diagnosed with cancer: a population-based cancer registration system and pathologic-based system. In the northern region of Iran, this process of collecting and analyzing different reports related to patients with cancer has an older history than other parts of this country. A population-based cancer registry in this region revealed that the incidence of upper gastrointestinal (GI) cancers in the North of Iran might be considered as the highest in the world (11). 

Based on the high prevalence, incidence and mortality rate related to gastrointestinal cancers in the northern region of Iran and to investigate the epidemiologic aspects of these neoplasms, this research was conducted to have a 10-year data analysis. 

## Methods

This observational analytical study was carried out based on the data collected by the Cancer Registration Center affiliated to the Vice Chancellery for Health of Babol University of Medical Sciences, North of Iran. Data related to malignancies of digestive organs which have been collected through a population-based cancer registry system from different sources including hospitals, medical clinics, family physicians, and laboratories during 2008-2017 have been classified and analyzed. 

Demographic and pathologic characteristics including age, gender, place of residence, and international classification of diseases (ICD-10) reference code have been recorded for each patient, diagnosed with digestive system cancer. To detect and exclude the repeated cases, the patients' national identification code has been checked. Crude incidence rate, and age-standardized incidence rate (ASR) have been calculated for different GI cancers, based on the primary involved site. Age-standardized incidence rate has been calculated by the method of standardization which has been described as follows: 1. To have a reference population, we used the world standard population available from: https://www.who.int/healthinfo/paper31.pdf (12). 2. Population of Babol City divided in different age-groups has been collected from the data bank of Vice Chancellery for Health of Babol University of Medical Sciences. 3. Crude age-specific incidence rate of different malignancies has been calculated for each age-group. 4. ASR of the malignancies of each digestive organ has been calculated based on the formula as: ("i" refers to age-groups; "di" refers to crude number of disease; "yi" is representative of the number of person-years that are at risk of disease; and "wi" means the population of the ith age-group in reference population). Data analysis was performed using SPSS-22 and excel-16 software package. ANOVA and chi-square test were used for data analysis. This research has been approved by the Ethics Committee of Babol University of Medical Sciences with the reference number IR.MUBABOL.HRI.REC.1398.270. 

## Results

**Figure 1:**
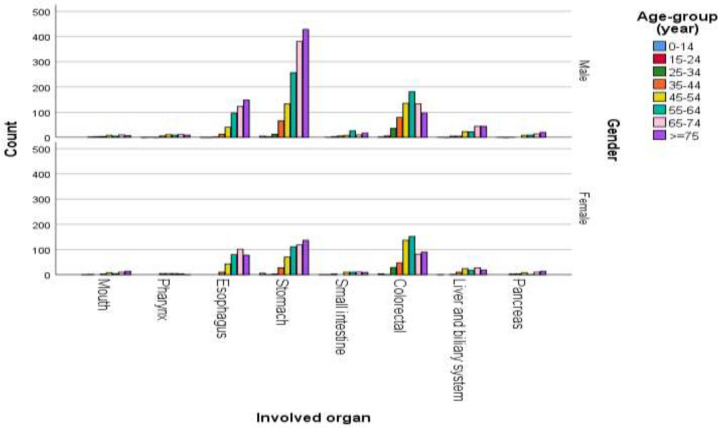
Crude number of different digestive system malignancies based on the patients' age-group and gender

**Figure 2:**
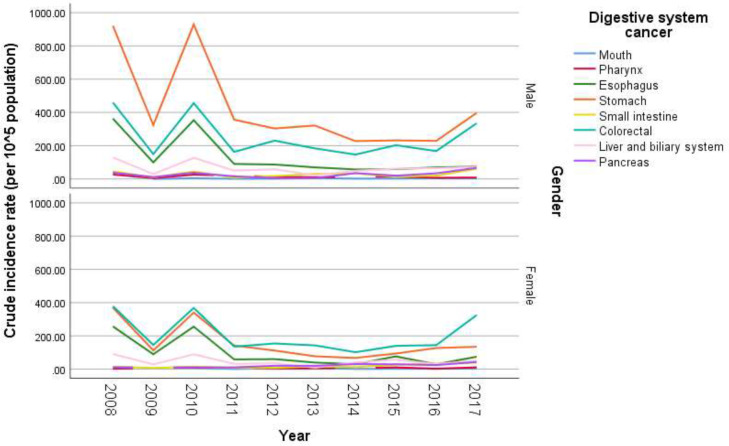
Crude incidence rate of different digestive system malignancies in two sexes, Babol, 2008-2017

Totally, 12660 records related to all types of malignancies from 2008 to 2017 have been reviewed. Among them, 4332 records (34.2%) with diagnosis of digestive system malignancies have been found. The average number of new cases with diagnosis of GI cancer in each year was 433.2, with a range of 237 newly diagnosed patients in year 2014 to 888 in year 2010. These patients had a range of age from less than 1 year to maximum age of 94 (mean age of 63.48±14.73) years. Most of GI cancers (2743; 63.3%) have been occurred in men, and 1589 (36.7%) in women (p<0.001). Crude number of annual new cases based on the involved site and the patients' gender has been summarized in table 1. This table shows that the most incident malignancies of digestive system have been from the stomach, colorectal and esophagus in men; and colorectal, stomach and esophagus in women, respectively. These three (gastric, colorectal and esophageal) cancers accounted for 3725 (85.98%) of total malignancies of digestive system. Also, the mean age of patients in various types of GI cancers was statistically different (p<0.001); the mean age of patients with colorectal malignancies was significantly lower than other types of GI cancers. Furthermore, the patients with esophageal and gastric cancers had higher mean ages than other patients with digestive system malignancies. The occurrence of GI cancers was not statistically different between the rural and urban areas (P=0.220). Crude age-specific incidence rate (CR), ASR of all types of GI cancers, and CR and ASR of the three most incident cancers were presented in table 2. Also, the CR of these cancers in ten years divided in male and female genders has been shown in figures 1 and 2. Table 2 shows that most of these cancers have been diagnosed in the age-groups of ≥75 (CR: 826.42 in 100,000 population), 65-74 (CR: 552.74 in 100,000), and 55-64 years (CR: 305.49 in 100,000), respectively. 

The incidence rate of gastrointestinal cancers has increased with age. The mean age of patients in different GI cancers were as follows: oral cavity (60.67±19.40); pharyngeal (58.82±14.61); esophageal (67.48±11.93); gastric (66.06±14.05); small intestinal (60.36±16.57); colorectal (58.02±14.71); hepatobiliary (62.94±15.02) and pancreatic (65.35±16.86). 

**Table 1:** Crude number of new cases with malignancies of digestive system; Babol, north of Iran; 2008-2017

**Year**	**2008**		**2009**		**2010**		**2011**		**2012**		**2013**		**2014**		**2015**		**2016**		**2017**	
Involved site	M	F	M	F	M	F	M	F	M	F	M	F	M	F	M	F	M	F	M	F
Oral cavity	0	0	2	0	9	9	1	2	2	0	7	10	2	3	3	4	5	7	8	8
Pharynx	11	1	2	0	11	5	0	0	6	1	4	2	0	0	7	5	3	1	4	5
Esophagus	113	80	31	28	112	81	29	19	29	20	23	13	19	10	20	26	24	10	26	26
Stomach	269	108	95	33	276	101	108	43	95	35	100	24	71	21	74	30	74	41	130	44
Small intestine	12	4	2	2	12	4	2	2	5	2	9	5	7	3	3	7	5	7	14	9
Colorectal	117	96	38	37	118	95	43	36	63	42	50	39	40	28	56	39	47	41	96	93
Liver, Gall bladder, and Biliary system	27	19	6	6	27	19	11	7	13	8	5	2	11	9	14	13	15	7	17	12
Pancreas	7	2	2	0	7	2	3	2	1	4	1	4	7	6	4	6	7	5	14	9
Total	866		284		888		308		326		298		237		311		299		515	

**Table 2:** Crude age-specific incidence rate (CR) and ASR of digestive system malignancies at Babol, north of Iran. 2008-2017

**Type**	**Year**	**2008**	**2009**	**2010**	**2011**	**2012**	**2013**	**2014**	**2015**	**2016**	**2017**
	**Age-group/CR in 10** ^5 ^ ** population**										
All types	0-14	5.99	2.06	2.04	1.00	0.91	0.98	0.98	1.89	2.82	2.79
	15-24	2.72	1.91	3.92	0	0	0	0	3.31	1.14	5.85
	25-34	26.42	10.45	25.88	4.33	6.25	9.26	7.15	5.88	5.73	10.25
	35-44	90.01	32.42	88.88	24.26	27.92	22.06	16.75	20.09	20.95	42.37
	45-54	278.00	94.33	257.13	81.94	95.03	73.12	63.23	76.32	64.63	128.19
	55-64	728.71	199.27	672.56	188.39	255.06	248.58	168.21	218.29	159.08	332.05
	65-74	1235.78	383.50	1278.28	406.11	384.97	370.71	299.91	324.25	297.37	576.16
	75+	1770.22	559.21	1599.43	785.24	639.31	516.13	422.03	632.78	730.93	842.25
	ASR	1834.33	552.17	1754.07	620.06	621.80	568.49	434.40	555.54	521.40	854.38
	95% CI for ASR	1637.36-2031.29	493.23-611.11	1563.84-1944.30	551.49-688.62	555.02-688.59	506.66-630.33	387.53-481.27	495.63-615.46	462.79-580.00	762.78-945.97
Gastric cancer	0-14	4.99	1.03	2.04	0	0.91	0.98	0	0	0	0.93
	15-24	0.91	0	0.98	0	0	0	0	0	0	2.34
	25-34	2.40	2.32	2.25	3.25	1.04	1.03	1.02	0.98	0.96	1.86
	35-44	31.94	14.10	30.55	8.09	10.64	9.08	5.15	3.77	3.70	10.89
	45-54	82.76	23.58	73.74	34.60	26.40	23.81	19.97	19.08	20.00	41.74
	55-64	282.56	81.84	259.47	77.96	75.57	69.72	70.83	79.38	63.08	124.85
	65-74	625.65	210.14	623.68	215.91	162.09	182.82	99.97	119.73	133.82	172.35
	75+	947.07	312.50	843.05	434.55	364.26	301.08	218.05	242.34	313.26	316.64
	ASR	863.08	277.37	809.07	314.85	262.82	248.84	180.23	204.67	220.31	296.59
	95% CI for ASR	768.04-958.12	246.42-308.31	719.04-899.10	278.52-351.17	233.04-292.60	220.86-276.81	160.46-200.01	182.18-227.16	195.00-245.61	264.60-328.58
Esophageal cancer	0-14	0	0	0	0	0	0	0	0	0	0
	15-24	0	0	0	0	0	0	0	0	0	0
	25-34	0	0	0	0	1.04	0	0	0.98	0	0
	35-44	8.71	2.82	8.33	0	1.33	0	1.29	1.26	2.47	4.84
	45-54	48.81	13.76	43.49	5.46	10.56	5.10	6.66	4.78	10.77	5.96
	55-64	189.61	64.05	174.12	25.98	34.64	33.35	14.76	17.01	10.97	29.22
	65-74	330.92	78.80	329.88	77.11	55.72	66.02	44.99	69.84	34.69	59.09
	75+	416.00	139.80	370.31	167.72	141.24	64.52	63.30	141.37	91.37	132.99
	ASR	462.17	138.52	434.15	113.39	102.29	82.06	55.80	94.37	56.58	96.82
	95% CI for ASR	411.09-513.23	123.21-153.83	385.66-482.64	99.77-126.99	90.75-113.83	72.65-91.47	49.42-62.18	82.71-106.03	49.70-63.47	85.84-107.80
Colorectal cancer	0-14	0	1.03	0	0	0	0	0.99	0.95	1.88	0.93
	15-24	1.82	0.95	1.96	0	0	0	0	1.10	0	1.17
	25-34	20.41	6.97	19.13	1.08	2.08	6.18	4.09	2.94	1.91	6.52
	35-44	42.10	14.10	40.28	13.48	10.64	7.79	6.44	11.30	7.40	16.95
	45-54	116.72	47.16	103.99	27.31	42.24	25.51	16.64	33.39	26.16	53.66
	55-64	182.18	46.26	167.29	71.46	116.51	112.16	56.07	93.55	57.60	140.79
	65-74	170.63	57.79	170.09	71.97	70.91	71.09	89.97	64.85	69.39	216.67
	75+	238.98	74.01	212.73	129.60	74.34	71.68	77.37	94.24	169.68	208.98
	ASR	334.70	99.64	312.19	141.17	162.41	156.43	120.28	146.03	140.30	302.41
	95% CI for ASR	298.31-371.92	89.66-109.64	278.31-346.06	125.72-156.62	140.66-184.15	135.36-177.49	106.69-133.86	128.20-163.86	125.18-155.43	268.80-336.02

CR=Crude incidence rate (per 100000 population); ASR= Age-standardized incidence rate; CI=confidence interval; SD=standard deviation

## Discussion

This 10-year data analysis revealed that nearly 34% of all-type incident cancers was related to digestive system malignancies. Gastrointestinal malignancies have been represented as the most common group of neoplasms in the world (13). The American Cancer Society estimated 53,260 new cases of oral cavity and pharynx, and 333,680 of gastrointestinal cancers in the year 2020; that accounts 21.4% of total 1,806,590 estimated new cases of cancer in the USA (14). An assessment of global burden from the five common GI cancers revealed that 26% of the global cancer incidence and 35% of all cancer-related deaths was attributed to GI neoplasms (15). In Iran, GI cancers are among the most common malignancies (13). Previous study in Iran has demonstrated a trend of increasing incidence of gastrointestinal cancers in both genders in Iran from 2000 to 2010 (4). 

Gastric, esophageal and colorectal cancers have been the most incident malignancies among different types of digestive system cancers in this region and accounted for about 86% of all GI tract cancers. A previous study about the trends of gastrointestinal cancer incidence in Iran from 2001 to 2010 reported these three neoplasms as the most incident GI malignancies among Iranian men and women (4). Other studies represented a high incidence rate of gastric cancer in the northern provinces of Iran like Mazandaran (3, 16). A 25-year trend analysis of gastrointestinal cancers in northern Iran which has been conducted based on data related to patients referring to a cancer-treatment center from 1991 to 2016 demonstrated an increasing rate of stomach and colorectal and decreasing rate of esophageal cancer in northern Iran (17). A trend analysis of GI cancers (1991-2015) in a high risk area of GI cancers in China showed a similar result and represented the gastric, esophageal and colorectal cancers as the most common GI neoplasms in that region (8). Increased life expectancy, lifestyle behaviors, especially sedentary lifestyle and changes in dietary pattern, increased public awareness about screening methods of cancer, advances in diagnostic approaches, improved cancer registry system, increased exposure to some risk factors of GI cancers and also socioeconomic conditions in this region might justify the higher incidence rate of these three malignancies (4, 18). 

Mean age of patients in this study was about 63.5 year, and a significant difference was observed in mean age of patients with various types of GI cancers. Patients with colorectal cancers had the least mean age. Disparities in access to screening approaches and also health-promoting lifestyle behaviors can be named as contributing factors in this variation between different GI cancers (19); for example, an integrated screening program for secondary prevention of colorectal cancers in first-degree relatives of the affected individuals has been conducted in the health system of Babol for many years. 

Crude incidence rate of digestive system malignancies ranged from 47.28 (per 100,000 population) in year 2014 to 183.96 in year 2008; and age-standardized incidence rate had a wide range from 434.40 to 1834.33 per 10^5^ population. A previous study in Fars province, southern Iran reported the CR and ASR of GI cancers as 142.16 and 240.96 in men, and 86.82 and 149.57 in women, respectively without including the malignancies originated from oral cavity and pharynx (20). A 12-year trend analysis of the incidence of GI cancers in East Azerbaijan province, northwest of Iran (2004-2015) revealed that the ASR for colorectal cancer increased from 2.9 to 13.6 per 100,000 women and from 2.2 to 17.8 per 100,000 men during the study period. The ASR for gastric cancer increased from 10.5 to 13.5 in women and from 3.1 to 29.9 per 100,000 men. However, the ASR of esophageal cancer in both genders showed a declining trend (21). Our result represented a considerable difference with review articles on global epidemiology of gastric (22) and colorectal (19) cancers based on GLOBOCAN 2018 data that estimated the age-standardized incidence rate of gastric cancer as 8.5 and 15.9; and this measure for colorectal cancer was reported 11.1 and 16.0 per 100,000 in Iran for females and males, respectively. 

Considering the inherited genetic factors, different infections, and lifestyle behaviors as important factors that are linked to incidence of GI cancer (23, 24), these factors can be associated to high incidence rate of digestive system malignancies in this region. For example, a previous study that examined lifestyle behaviors of middle-aged (40-60 years old) adults in this region revealed that 44.80% of women and 46.30% of men had overweight (body mass index from 25 to 29.9 kg/m2); and 9.10% of women and 23.80% of men had obesity (body mass index ≥30 kg/m2) (25). 

The World Health Organization estimated that nearly 28% of adults aged 18 years and over were insufficiently physically active; and they engaged in less than 150 minutes of moderate or intensive physical activity per week (26); similar result has been reported about insufficient physical activity in middle-aged adults from this region (25). Furthermore, people in northern Iran have some particular dietary habits such as over-eating, consumption of high fat dairy products (milk, cheese and yogurt), high fat food, moldy food, pickled vegetables and intake of deep-fried animal-source products that can increase GI cancer risk (15, 27). A considerable portion of previous studies that examined GI cancers did not include oral and pharyngeal neoplasms; however, we included new cases of oral cavity and pharyngeal malignancies as digestive system cancers in this research because of some similarities in clinical manifestations and etiology of oropharyngeal cancers with upper esophageal malignancies (28), and it can be mentioned as a strong point of this study. The most important limitation of this research was considerable variation in incident gastrointestinal cancers which have been reported and registered in different years, though we cannot present a clear and precise reason for this variation.

 It might be due to some reasons; the crude number of new cases of cancer patients might be higher or lower in some years; or the data collection and registration system might have strong or weak points in different years; or the reporting sources might be different in different years. 

In conclusion, gastric, esophageal and colorectal cancers were the most prevalent digestive system malignancies in Babol, North of Iran, and accounted for about 86% of all GI tract cancers. A considerable variation has been found in incident gastrointestinal cancers in different years. 

## Funding:

This study has been supported by Babol University of Medical Sciences, Babol, Iran.

## Conflict of interests:

The authors declare that they have no competing interests**. **

## List of abbreviations

GI= Gastrointestinal 

ICD= International classification of diseases

ASR= Age-standardized incidence rate

CR= Crude incidence rate

## Ethics approval and consent to participate

This research has been approved by the Ethics Committee of Babol University of Medical Sciences with the reference number IR.MUBABOL.HRI.REC.1398.270. All patients whose related data were registered in the research had verbal informed consent to participate. A written informed consent was obtained from the hospitalized patients. 

## Availability of data and materials

Data supporting the results reported in the manuscript can be found via academic researches thru email to the corresponding author at **dr.mouodi@gmail.com. **

## Authors' contributions

LSA, NN, MJ and JSS had substantial contributions to conception and design, acquisition of data, analysis and interpretation of data.

SM had substantial contributions to conception and design, drafting the article and revising it critically for important intellectual content. 

TM, SA, FV, and MM contributed in acquisition of data. 

HS had substantial contribution in analysis and interpretation of data. 

All authors approved the final version of the manuscript to be published.
